# Deep Recurrent Neural Network-Based Autoencoders for Acoustic Novelty Detection

**DOI:** 10.1155/2017/4694860

**Published:** 2017-01-15

**Authors:** Erik Marchi, Fabio Vesperini, Stefano Squartini, Björn Schuller

**Affiliations:** ^1^Machine Intelligence & Signal Processing Group, Technische Universität München, Munich, Germany; ^2^audEERING GmbH, Gilching, Germany; ^3^Chair of Complex & Intelligent Systems, University of Passau, Passau, Germany; ^4^A3LAB, Department of Information Engineering, Università Politecnica delle Marche, Ancona, Italy; ^5^Department of Computing, Imperial College London, London, UK

## Abstract

In the emerging field of acoustic novelty detection, most research efforts are devoted to probabilistic approaches such as mixture models or state-space models. Only recent studies introduced (pseudo-)generative models for acoustic novelty detection with recurrent neural networks in the form of an autoencoder. In these approaches, auditory spectral features of the next short term frame are predicted from the previous frames by means of Long-Short Term Memory recurrent denoising autoencoders. The reconstruction error between the input and the output of the autoencoder is used as activation signal to detect novel events. There is no evidence of studies focused on comparing previous efforts to automatically recognize novel events from audio signals and giving a broad and in depth evaluation of recurrent neural network-based autoencoders. The present contribution aims to consistently evaluate our recent novel approaches to fill this white spot in the literature and provide insight by extensive evaluations carried out on three databases: A3Novelty, PASCAL CHiME, and PROMETHEUS. Besides providing an extensive analysis of novel and state-of-the-art methods, the article shows how RNN-based autoencoders outperform statistical approaches up to an absolute improvement of 16.4% average *F*-measure over the three databases.

## 1. Introduction

Novelty detection aims at recognizing situations in which unusual events occur. The challenging task of novelty detection is usually considered as single class classification task. The “normal” data traditionally comprises a very big set which allows for an accurate modelling. The acoustic events not included in the “normal” data are treated as* novel* events. Novel patterns are tested by comparing them with the normal class model resulting in a novelty score. Then, the score is processed by a decision logic—typically a threshold—to decide whether the test sample is novel or normal.

A plethora of approaches have been proposed due to the practical relevance of novelty detection, especially for medical diagnosis [1–3], damage inspection [4, 5], physiological condition monitoring [6], electronic IT security [7], and video surveillance systems [8].

According to [9, 10], novelty detection techniques can be grouped into two macro categories: (i) statistical and (ii) neural network-based approaches. Extensive studies have been made in the category of statistical and probabilistic approaches which are evidently the most widely used in the field of novelty detection. The approaches on this category are modelling data based on its statistical properties and exploiting this information to determine when an unknown test sample belongs to the learnt distribution or not. Statistical approaches have been applied to a number of applications [9] ranging from data stream mining [11], outlier detection of underwater targets [12], the recognition of cancer [1], nondestructive inspection for the analysis of mechanical components [13], and audio segmentation [14], to many others. In 1999, support vector machines (SVMs) were introduced in the field of novelty detection [15] and subsequently applied to time-series [16, 17], jet engine vibration analysis [18], failure detection in jet engines [19], patient vital-sign monitoring [20], fMRI analysis [21], and damage detection of a gearbox wheel [22].

Neural network-based approaches—also named reconstruction-based [23]—have gained interest in recent years along with the evident success of neural networks in several other fields. In the past decade, several works focused on the application of a neural network in the form of an autoencoder (AE) have been presented [10], given the huge impact and effectiveness of neural networks. The autoencoder-based approaches involve building a regression model using the “normal” data. The test data are processed by analysing the reconstruction error between the regression target and the encoded value. When the reconstruction error shows high score, the test data is considered novel. Examples of applications include such to detect abnormal CPU data usage [24, 25] and such to detect outliers [26–29] for damage classification under changing environmental conditions [30].

In these scenarios, very little studies have been conducted in the field of acoustic novelty detection. Recently, we observed a growing research interest in application domains involving surveillance and homeland security to monitor public places or supervise private environments where people may live alone. Driven by the increasing requirement of security, public places such as but not limited to stores, banks, subway, trains, and airports have been equipped with various sensors like cameras or microphones. As a consequence, unsupervised monitoring systems have gained much attention in the research community to investigate new and efficient signal processing approaches. The research in the area of surveillance systems mainly focusses on detecting abnormal events relying on video information [8]. However, it has to be noted that several advantages can be obtained by relying on acoustic information. In fact, acoustic signals—as opposed to video information—need low computational costs and are invariant to illumination conditions, possible occlusion, and abrupt events (e.g., a shotgun and explosions). Specifically in the field of acoustic novelty detection, studies focused only on statistical approaches by applying hidden Markov models (HMM) and Gaussian mixture models (GMM) to acoustic surveillance of abnormal situations [31–33] and to automatic space monitoring [34]. Despite the number of studies exploring statistical and probabilistic approaches, the use of neural network-based approaches for acoustic novelty detection has only been introduced recently [35, 36].

*Contribution*. Only in the last two years the use of neural networks for acoustic novelty detection has gained interest in the research community. In fact, few recent studies proposed a (pseudo-)generative model in the form of a denoising autoencoder with recurrent neural networks (RNNs). In particular, the use of Long-Short Term Memory (LSTM) RNNs as generative model [37] was investigated in the field of text generation [38], handwriting [38], and music [39]. However, the use of LSTM as a model for audio generation was only introduced in our recent works [35, 36].

This article provides a broad and extensive evaluation of state-of-the-art methods with a particular focus on novel and recent unsupervised approaches based on RNN-based autoencoders. We significantly extended the studies conducted in [35, 36] by evaluating further approaches such as one-class SVMs (OCSVMs) and multilayer perceptrons (MLP), and most importantly we conducted a broad and in depth evaluation on three different datasets for a total number of 160 153 experiments, making this article the first to present such a complete evaluation in the field of acoustic novelty detection.

We evaluate and compare all these methods with three different databases: A3Novelty, PASCAL CHiME, and PROMETHEUS. We provide evidence that RNN-based autoencoders significantly outperform other methods by outperforming statistical approaches up to an absolute improvement of 16.4% average *F*-measure over the three databases.

The remainder of this contribution is structured as follows: First, a basic description of the different statistical methods is given in Section 2. Then, the feed-forward and LSTM RNNs together with autoencoder-based schemes for acoustic novelty detection are described (Sections 3 and 4). Next the thresholding strategy and features employed in the experiments are given in Section 5. The used databases are introduced in Section 6 and the experimental set-up is discussed in Section 7 before discussing the evaluation of obtained results in Section 8.  Section 9 finally presents our conclusions.

## 2. Statistical Methods

In this section we introduce statistical approaches such as GMM, HMM, and OCSVM. We formally define the input vector *x* ∈ **R**^*n*^, where *n* is the number of acoustic features (cf. Section 5).

### 2.1. Gaussian Mixture Models

GMMs estimate the probability density of the “normal” class, given training data, using a number of Gaussian components. The training phase of a GMM exploits the *k*-means algorithm or other suited training algorithms and the Expectation-Maximisation (EM) algorithm [40]. The former initializes the parameters while *L* iterations of EM algorithm lead to the final model. Given a predefined threshold (defined in Section 5), if the probability produced by the GMM with a test sample is lower than the threshold, the sample is detected as novel event.

### 2.2. Hidden Markov Models

A further statistical model is the HMM [41]. HMMs differ from GMMs in terms of input temporal evolution. Indeed, while a diagonal GMM tends to approximate the whole training data probability distribution by means of a number of Gaussian components a HMM models the variations of the input signal through its hidden states. The HMM topology employed in this work is* left-right* and it is trained by means of the* Baum-Welch* algorithm [41] while regarding the novelty detection phase, the decision is based on the* sequence* paradigm. Considering a left-right HMM having *N*_*s*_ hidden states, a* sequence* is a set of *N*_*s*_ feature vectors: $\tilde{x}=\{x_{1},\ldots ,x_{N_{s}}\}$. The emission probabilities of these observable events are determined by a probability distribution, one for each state [9]. We trained an HMM on what we call “normal” material and exploited the log-likelihoods as novelty scores. In the testing phase, the unseen signal is segmented into a fixed length depending on the number of states of the HMM, and if the log-likelihood is higher than the defined threshold (cf. Section 5), the segment is detected as novel.

### 2.3. One-Class Support Vector Machines

A OCSVM [42] maps an input example onto a high-dimensional feature space and iteratively searches for the hyperplane that maximises the distance between the training examples from the origin. In this constellation, the OCSVM can be seen as a two-class SVM where the origin is the unique member of the second class, whereas the training examples belong to the first class. Given the training data *x*_1_,…, *x*_*l*_ ∈ *X*, where *l* is the number of observations, the class separation is performed by solving the following:

(1) minw,ξ,ρ 12w21νl∑iξi−ρ,subject  to: w·Φxi≥ρ−ξi,ξi≥0,

where *w* is the support vector, *ξ*_*i*_ are slack variables, *ρ* is the offset, and Φ maps *X* into a dot product space *F* such that the dot product in the image of Φ can be computed by evaluating a certain kernel function such as a linear or Gaussian radial base function:

(2) $$\begin{array}{c} k\left(\;x,y\;\right)=\exp\left(\;-\frac{{\left\|x-y\right\|}^{2}}{2\sigma^{2}}\;\right). \end{array}$$

The parameter *ν* sets an upper bound on the fraction of the outliers defined to be the data being outside the estimated region of normality. Thus, the decision values are obtained with the following function:

(3) $$\begin{array}{c} f\left(\;x\;\right)=w\cdot \Phi \left(\;x\;\right)-\rho . \end{array}$$

We trained a OCSVM on what we call “normal” material and used the decision values as novelty scores. During testing, the OCSVM provides a decision value for the unseen pattern, and if the decision value is higher than the defined threshold (cf. Section 5), the segment is detected as novel.

## 3. Feed-Forward and Recurrent Neural Networks

This section introduces the MLP and the LSTM RNNs employed in our acoustic novelty detectors.

The first neural network type we used is a multilayer perceptron [43]. In a MLP the units are arranged in layers, with feed-forward connections from one layer to the next. Each node outputs an activation function applied over the weighted sum of its inputs. The activation function can be linear, a hyperbolic function (tanh) or the sigmoid function. Input examples are fed to the input layer, and the resulting output is propagated via the hidden layers towards the output layer. This process is known as the forward pass of the network. This type of neural networks only relies on the current input and not on any past or future inputs.

**Figure 1:**
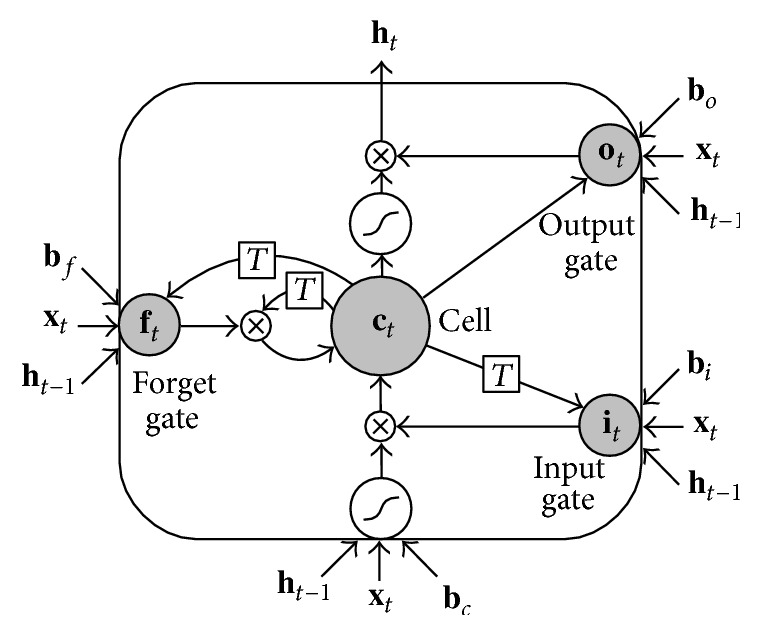
LSTM unit, comprising the input, output, and forget gates and the memory cell.

The second neural network type we employed is the LSTM RNN [44]. Compared to a conventional RNN, the hidden units are replaced by so-called memory blocks. These memory blocks can store information in the “cell variable” **c**_*t*_. In this way, the network can exploit long-range temporal context. Each memory block consists of a memory cell and three gates: the input gate, output gate, and forget gate, as depicted in Figure 1.

The memory cell is controlled by the input, output, and forget gates.

The stored cell variable **c**_*t*_ can be reset by the forget gate, while the functions responsible for reading input from **x**_*t*_ and writing output to **h**_*t*_ are controlled by the input and output gates, respectively:

(4) ct=ft⊗ct−1+it⊗tanh⁡Wxcxt+Whcht−1+bc,ht=ot⊗tanh⁡ct,

where tanh and ⊗ stand for element-wise hyperbolic tangent and element-wise multiplication, respectively. The output of the input gates is denoted by the variable **i**_*t*_, while the output of the output and forget gates are indicated by **o**_*t*_ and **f**_*t*_, respectively. The variable **W** denotes a weight matrix, and **b**_*c*_ indicates a bias term.

Each LSTM unit is a separate and independent block. In fact, the size of **h**_*t*_ is the same as **i**_*t*_, **o**_*t*_, **f**_*t*_, and **c**_*t*_. The size corresponds to the number of LSTM units in the hidden layer. In order to have the gates being dependent uniquely from the memory cell within the same LSTM unit, the matrices of the weights from the cells to the gates are diagonal.

Furthermore, we employed bidirectional RNN (BRNN) [45], which are capable of learning the context in both temporal directions. In fact, a BRNN contains two distinct hidden layers, which are processing the input vector in each direction. The output layer is then connected to both hidden layers. A more complex architecture can be obtained by combining a LSTM unit with a BRNN, which is referred to as bidirectional LSTM (BLSTM) [46]. BLSTM exploits context from both temporal directions. Note that, in the case of BLSTM, it is not possible to perform online processing as a short buffer to look ahead is required.

When the layout of a neural network comprises more hidden layers, it is defined as deep neural network (DNN) [47]. An incrementally higher level representation of the input data is provided when multiple hidden layers are stacked on each other (deep learning).

In the case of multiple layers, the output of a BRNN is computed as

(5) $$\begin{array}{c} y_{t}=W_{\vec{h^{N}}y}\vec{h_{t}^{N}}+W_{\overset{⟵}{h^{N}}y}\overset{⟵}{h_{t}^{N}}+b_{y}, \end{array}$$

where the forward and backward activation of the *N*th (last) hidden layer are denoted by $\vec{h_{t}^{N}}$ and $\overset{⟵}{h_{t}^{N}}$, respectively. The reconstructed signal is generated by using an identity activation function at the output. The best network layout was obtained by conducting a number of preliminary evaluations. Several configurations were evaluated by changing the size and the number of hidden layers (i.e., the number of LSTM units for each layer).

The training procedure was iterated up to a maximum of 100 epochs. The standard gradient descent with backpropagation of the sum squared error was used to recursively update the network weights. Those were initialized with a random Gaussian distribution with mean 0 and standard deviation 0.1, as it usually provides an acceptable initialization in our experience.

## 4. Autoencoders for Acoustic Novelty Detection

This section introduces the concepts of autoencoders and describes the basic autoencoder, compression autoencoder, denoising autoencoder, and nonlinear predictive autoencoder [36].

### 4.1. Basic Autoencoder

A basic autoencoder is a neural network trained to set the target values equal to the inputs. Its structure typically consists of only one hidden layer, while the input and the output layers have the same size. The training set *𝒳*_tr_ consists of background environmental sounds, while test set *𝒳*_te_ is composed of recordings containing abnormal sounds. It is used to find common data representation from the input [48, 49]. Formally, in response to an input example *x* ∈ **R**^*n*^, the hidden representation *h*(*x*) ∈ **R**^*m*^ is

(6) $$\begin{array}{c} h\left(\;x\;\right)=f\left(\;W_{1}x+b_{1}\;\right), \end{array}$$

where *f*(*z*) is a nonlinear activation function, typically a logistic sigmoid function *f*(*z*) = 1/(1 + exp⁡(−*z*)) applied componentwisely, *W*_1_ ∈ **R**^*m*×*n*^ is a weight matrix, and *b*_1_ ∈ **R**^*m*^ is a bias vector.

The network output maps the hidden representation *h* back to a reconstruction $\tilde{x}\in R^{n}$:

(7) $$\begin{array}{c} \tilde{x}=f\left(\;W_{2}h\left(\;x\;\right)+b_{2}\;\right), \end{array}$$

where *W*_2_ ∈ **R**^*n*×*m*^ is a weight matrix and *b*_2_ ∈ **R**^*n*^ is a bias vector.

Given an input set of examples *𝒳*, AE training consists in finding parameters *θ* = {*W*_1_, *W*_2_, *b*_1_, *b*_2_} that minimise the reconstruction error, which corresponds to minimising the following objective function:

(8) $$\begin{array}{c} J\left(\;\theta \;\right)=\underset{x\in X}{\sum }{\left\|\;x-\tilde{x}\;\right\|}^{2}. \end{array}$$

**Figure 2:**
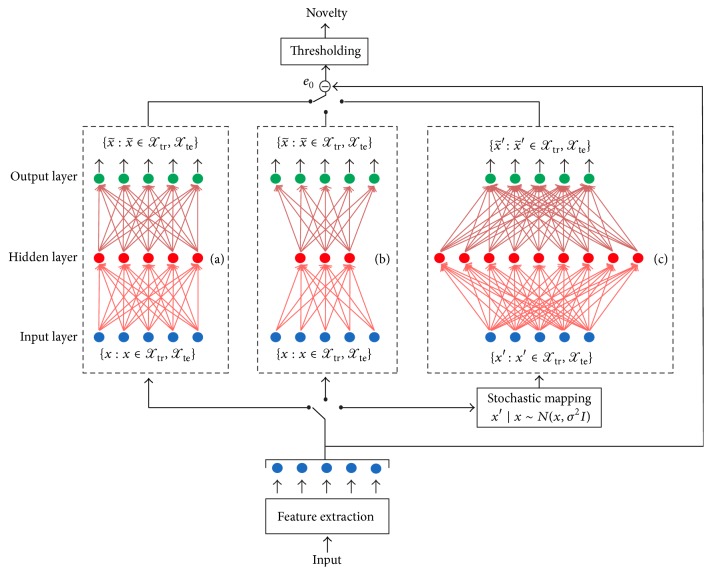
Block diagram of the proposed acoustic novelty detector with different autoencoder structures. Features are extracted from the input signal and the reconstruction error between the input and the reconstructed features is then processed by a thresholding block which detects the* novel* or* nonnovel* event. Structure of the (a) basic autoencoder, (b) compression autoencoder, and (c) denoising autoencoder on the training set *𝒳*_tr_ or testing set *𝒳*_te_. *𝒳*_tr_ contains data of nonnovel acoustic events; *𝒳*_te_ consists of* novel* and* nonnovel* acoustic events.

A well-known approach to minimise the objective function is the stochastic gradient descent with error backpropagation. The layout of the AE is shown in Figure 2(a).

### 4.2. Compression Autoencoder

The compression autoencoder (CAE) learns a compressed representation of the input when the number of hidden units *m* is smaller than the number of input units *n*. For example, if some of the input features are correlated, these correlations are learnt and reconstructed by the CAE. The structure of the CAE is given in Figure 2(b).

### 4.3. Denoising Autoencoder

In the denoising AE (DAE) [50] configuration the network is trained to reconstruct the original input from a corrupted version of it. The initial input *x* is corrupted by means of additive isotropic Gaussian noise in order to obtain *x*′∣*x* ~ *N*(*x*, *σ*^2^*I*). The corrupted input *x*′ is then mapped, as with the AE, to a hidden representation

(9) $$\begin{array}{c} h\left(\;x^{\prime }\;\right)=f\left(\;W_{1}^{\prime }x^{\prime }+b_{1}^{\prime }\;\right), \end{array}$$

forcing the hidden layer to retrieve more robust features and prevent it from simply learning the identity. Thus, the original signal is reconstructed as follows:

(10) $$\begin{array}{c} {\tilde{x}}^{\prime }=f\left(\;W_{2}^{\prime }x+b_{2}^{\prime }\;\right). \end{array}$$

The structure of the denoising autoencoder is shown in Figure 2(c). In the training phase, the set of network weights and biases *θ*′ = {*W*_1_′, *W*_2_′, *b*_1_′, *b*_2_′} are updated in order to have ${\tilde{x}}^{\prime }$ as close as possible to the uncorrupted input *x*. This procedure corresponds to minimising the reconstruction error objective function (8). In our approach, to corrupt the initial input *x*_*t*_ we make use of additive isotropic Gaussian noise, in order to obtain *x*′∣*x* ~ *N*(*x*, *σ*^2^*I*).

### 4.4. Nonlinear Predictive Autoencoder

The basic idea of a nonlinear predictive (NP) AE is to train the AE in order to predict the current frame from a previous observation. Formally, the input up to a given time frame *x*_*t*_ is mapped to a hidden representation *h*:

(11) $$\begin{array}{c} h\left(\;x_{t}\;\right)=f\left(\;W_{1}^{*},b_{1}^{*},x_{1,\ldots ,t}\;\right), \end{array}$$

where *W* and *b* denote weights and bias, respectively. From this, we reconstruct an approximation of the original signal as follows:

(12) $$\begin{array}{c} {\tilde{x}}_{t+k}=f\left(\;W_{2}^{*},b_{2}^{*},h_{1,\ldots ,t}\;\right), \end{array}$$

where *k* is the prediction delay and *h*_*i*_ = *h*(*x*_*i*_). A prediction delay of *k* = 1 corresponds to a shift of 10 ms in the audio signal in our setting (cf. Section 5). The training of the parameters is performed by minimising the objective function (8)—the difference is that $\tilde{x}$ is now based on nonlinear prediction according to (11) and (12). Thus, the parameters *θ*^*∗*^ = {*W*_1_^*∗*^, *W*_2_^*∗*^, *b*_1_^*∗*^, *b*_2_^*∗*^} are trained to minimise the average reconstruction error over the training set, to have ${\tilde{x}}_{t+k}$ as close as possible to the prediction delay. The resulting structure of the nonlinear predictive denoising autoencoder (NP-DAE) is similar to the one depicted in Figure 2(c), but with input and output updated as described above.

## 5. Thresholding and Features

This section describes the thresholding decision strategy and the features employed in our experiments.

### 5.1. Thresholding

Auditory spectral features (ASF) in Section 5.2 used in this work are composed by 54 coefficients, which means that the input and output layer of the network have 54 units each. The trained AE reconstructs each sample and novel events are identified by processing the reconstruction error signal with an adaptive threshold. The input audio signal *x* is segmented into sequences of 30 seconds of length. In the testing phase, we compute—on a frame basis—the average Euclidean distance between the networks' outputs and each standardized input feature value. In order to compress the reconstruction error to a single value, the distances are summed up and divided by the number of coefficients. Then we apply a threshold *θ*_th_ to obtain a binary signal, shifting from the median of the error signal of a sequence *e*_0_ by a multiplicative coefficient *β*. The coefficient ranges from *β*_min_ = 1 to *β*_max_ = 2:

(13) $$\begin{array}{c} \theta_{th}=\beta *median\left(\;e_{0}\left(\;1\;\right),\ldots ,e_{0}\left(\;N\;\right)\;\right). \end{array}$$

**Figure 3:**
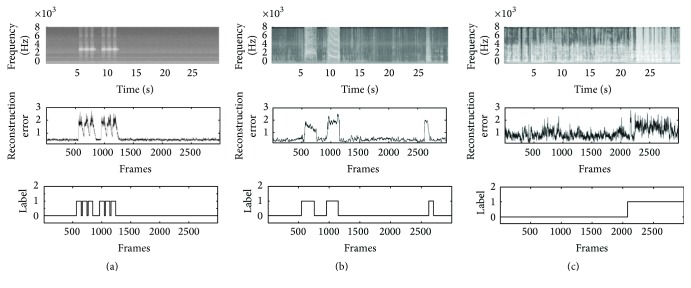
Spectrogram of a 30-second sequence from (a) the A3Novelty Corpus containing a novel siren event (top), (b) the PASCAL CHiME database containing three novel events, such as a siren and two screams, and (c) the PROMETHEUS database containing a novel event composed by an alarm and people screaming together (top). Reconstruction error signal of the related sequence obtained with a BLSTM-DAE (middle). Ground-truth binary novelty signal: novel (0), and not-novel (0) (bottom).

Figure 3 shows the reconstruction error for a given sequence. The figure clearly depicts a low reconstruction error in reproducing normal input such as talking, television sounds, and other normal environmental sounds.

### 5.2. Acoustic Features

An efficient representation of the audio signal can be achieved by extracting the auditory spectral features (ASF) [51]. The audio signal is split into frames with the size equal to 30 ms and a frame step of 10 ms, and then the ASF are obtained by applying Short Time Fourier Transform (STFT), which yields the power spectrogram of the frame. Mel spectrograms *M*_30_(*n*, *m*) (with *n* being the frame index and *m* the frequency bin index) are calculated converting the power spectrogram to the Mel-frequency scale using a filter-bank with 26 triangular filters. A logarithmic scaling is chosen to match the human perception of loudness:

(14) $$\begin{array}{c} M_{log}^{30}\left(\;n,m\;\right)=\log\left(\;M_{30}\left(\;n,m\;\right)+1.0\;\right). \end{array}$$

In addition, the positive first-order differences *D*_30_(*n*, *m*) are calculated from each Mel spectrogram following

(15) $$\begin{array}{c} D_{30}\left(\;n,m\;\right)=M_{log}^{30}\left(\;n,m\;\right)-M_{log}^{30}\left(\;n-1,m\;\right). \end{array}$$

Furthermore, the frame energy and its derivative are also included as feature ending up in a total number of 54 coefficients. For better reproducibility, the features extraction process is computed with our open-source audio analysis toolkit openSMILE [52].

## 6. Databases

This section describes the three databases evaluated in our experiments: A3Novelty, PASCAL CHiME, and PROMETHEUS.

### 6.1. A3Novelty

The A3Novelty Corpus (http://www.a3lab.dii.univpm.it/research/a3novelty) includes around 56 hours of recording acquired in a laboratory of the Università Politecnica delle Marche. These recordings were performed during different day and night hours, so very different acoustic conditions are available. A variety of* novel* events were randomly played back by a speaker (e.g., scream, fall, alarm, or breakage of objects) during the recordings.

Eight microphones were used in the recording room for the acquisitions: four Behringer B-5 microphones with cardioid pattern and an array of four AKG C400 BL microphones spaced by 4 cm, and then A MOTU 8pre sound card and the NU-Tech software were utilised to record the microphone signals. The sampling rate was equal to 48 kHz.

The abnormal event sounds (cf.  Table 1) can be grouped into four categories and they are freely available to download from http://www.freesound.org/:

- *Sirens*, three different types of sirens or alarm sounds.
- *Falls*, two occurrences of a person or an object falling to the ground.
- *Breakage of objects*, noise produced by the breakage of an object after the impact with the ground.
- *Screams*, four different human screams, both produced by a single person or by a group of people.

The A3Novelty Corpus is composed of two types of recordings:* background*, which contains only background sounds such as human speech, technical tools noise, and environmental sounds and* background with novelty*, which contains in addition to the background the artificially generated novelty events.

In the original A3Novelty database the recordings are segmented in sequences of 30 seconds. In order to limit the size of training data, we randomly selected 300 sequences from the* background* partition to compose training material (150 minutes) and 180 sequences from the* background with novelty* partition to compose the testing set (90 minutes). The test set contains 13 novelty occurrences.

For reproducibility, the list of randomly selected recordings and the train and test set are made available (http://www.a3lab.dii.univpm.it/research/a3novelty).

### 6.2. PASCAL CHiME

The original dataset is composed of around 7 hours of recordings of a home environment, taken from the PASCAL CHiME speech separation and recognition challenge [53]. It consists of a typical in-home scenario (a living room), recorded during different days and times, while the inhabitants (two adults and two children) perform common actions, such as talking, watching television, playing, or eating. The dataset was recorded with a binaural microphone and a sample-rate of 16 kHz. In the original PASCAL CHiME database the recordings are segmented in sequences of 5 minutes' duration. In order to limit the size of training data, we randomly selected sequences to compose 100 minutes of background for the training set and around 70 minutes for the testing set. For reproducibility, the list of randomly selected recordings and the train and test set are made available (http://a3lab.dii.univpm.it/webdav/audio/Novelty_Detection_Dataset.tar.gz). The test set was generated adding different types of sounds (taken from http://www.freesound.org/), such as screams, alarms, falls, and fractures (cf.  Table 1), after their normalization to the volume of the background recordings. The events in the test set were added at random position; thus the distance between one event and another is not fixed.

### 6.3. PROMETHEUS

The PROMETHEUS database [31] contains recordings of various scenarios designed to serve a wide range of real-world applications. The database includes (1) a* smart-room* indoor home environment including phases where a user is interacting with an automated speech-driven home assistant and (2) an outdoor public space consisting of (a) interaction of people with an* ATM*, (b) an* outdoor* security scenario in which people are waiting in front of a counter, and (3) an indoor office* corridor* scenario for security monitoring in standard indoor space. These scenarios substantially differ in terms of acoustic environment. The indoor scenarios were recorded under quiet acoustic conditions, whereas the outdoor recordings were conducted in an open-air public area and contain nonstationary background noise. The smart-home scenario contains recordings of five professional actors performing five single-person and 14 multiple-person action scripts. The main activities include human-machine interaction with a virtual home agent and a number of alternating normal and abnormal activities specifically designed to monitor and interpret human behaviour. The single-person and multiple-person actions include abnormal events, such as falls, alarm followed by panic, atypical vocalic reactions (pain, fear, and anger), or fractures. Examples are walking to the couch, sitting, or interacting with the smart environment to turn the TV on, open the windows, or decrease the temperature. The scenarios were recorded three to five times, by changing the actors and their roles in the action scripts.  Table 1 provides details on the number of abnormal events per scenario, including average time duration.

## 7. Experimental Set-Up

The networks were trained with the gradient steepest descent algorithm on the sum of squared errors (SSE). In the case of all the LSTM and BLSTM networks, we used a constant value of learning rate *l* = 1*e*^−6^ since it showed better performances in our previous works [35], whereas different values of *l* = {1*e*^−8^, 1*e*^−9^} were used for MLP networks. Different noise sigma values *σ* = {0.01,0.1,0.25} were applied to the DAE. No Gaussian noise was applied to the basic AE and to the CAE following the architectures described in Section 4. The prediction delay was applied for different values: *k* = {1,2, 3,4, 5,…, 10}. The AEs were trained using our open-source CUDA RecurREnt Neural Network Toolkit (CURRENNT) [54] ensuring reproducibility. As evaluation metrics we used *F*-measure in order to compare the results with previous works [35, 36]. We evaluated several topologies for the nonlinear predictive DAE ranging from 54-128-54 to 216-216-216 and from 54-30-54 to 54-54-54 in the case of CAE and basic AE, respectively. Every network topology was evaluated for each of the 100 epochs of training. In order to compare our results with our previous studies we kept the same optimisation procedure as applied in [35, 36]. We employed further three state-of-the-art approaches based on OCSVM, GMM, and HMM. In the case of OCSVM, we trained models at different complexity values *C* = {0.05,0.01,0.005,0.001,0.0005,0.0001}. Radial basis function kernels were used with different gamma values *γ* = {0.01,0.001}, and we controlled the fraction of outliers in the training phase with different values *ν* = {0.1,0.01}. The OCSVM was trained via the LIBSVM library [55]. In the case of GMM, models were trained at different numbers of Gaussian components 2^*n*^ with *n* = {1,2,…, 8}, whereas left-right HMMs were trained with different numbers of states *s* = {3,4, 5} and 2^*n*^ Gaussian components with *n* = {1,2,…, 7}. GMMs and HMMs were trained using the* Torch* [56] toolkit. The decision values produced as output of the OCSVM and the probability estimates produced as output of the probabilistic models were postprocessed with a similar thresholding algorithm (cf. Section 5) in order to fairly compare the performance among the different methods. For all the experiments and settings we maintained the same feature set.

## 8. Results

In this section we present and comment on the results obtained in our evaluation across the three databases.

### 8.1. A3Novelty

Evaluations on the A3Novelty Corpus are reported in the second column of Table 2. In this dataset GMMs and HMMs perform similarly; however, they are outperformed by the OCSVM with a maximum improvement of 3.6% absolute *F*-measure. The autoencoder-based approaches are significantly boosting the performance up to 98.7%. We observe a vast absolute improvement by up to 6.9% against the probabilistic approaches. Among the three CAE, AE, and DAE, we observe that compression and denoising layouts with BLSTM units perform closely to each other at up to 98.7% in the case of the BLSTM-CAE. This can be due to the fact that the dataset contains fewer variations in the background material used for training, and the feature selection operated internally by the AE increases the sensitivity of the reconstruction error.

The nonlinear predictive results are shown in the last part of Table 2. We provide performance in the three named configurations and with the three named unit types. In concordance to what we found in the PASCAL database, the NP-BLSTM-DAE method provided the best performance in terms of *F*-measure of up to 99.4%. A significant absolute improvement (one-tailed* z*-test [57], *p* < 0.01 (in the rest of the manuscript we reported as “significant” the improvements with at least *p* < 0.01 under the one-tailed* z*-test [57])) of 10.0%  *F*-measure is observed against the GMM-based approach, while an absolute improvement of 7.6%  *F*-measure is exhibited with respect to the OCSVM method. We observe an overall improvement of ≈1% between the “ordinary” and the “predictive” architectures.

**Figure 4:**
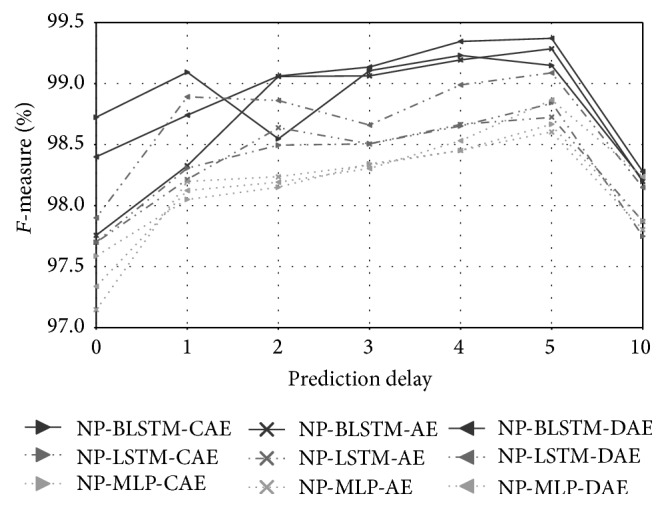
A3Novelty: performances obtained with NP-Autoencoders by progressively increasing the prediction delay.

The performance obtained by progressively increasing the prediction delay (*k*) values (from 0 up to 10) is reported in Figure 4. We evaluated the compression autoencoder (CAE), the basic autoencoder (AE), and the denoising autoencoder (DAE) with MLP, LSTM, and BLSTM units, and we applied different layouts (cf. Section 7) per network type. However, for the sake of brevity, we only show the best configurations. The best results across all the three unit types are 99.4% and 99.1%  *F*-measure for the NP-BLSTM-DAE and NP-LSTM-DAE networks, respectively. These are obtained with a prediction delay of 5 frames, which translates into an overall delay of 50 ms. In general, the best performances are achieved with *k* = 4 or *k* = 5. Increasing the prediction delay up to 10 frames produces a heavy decrease in performance down to 97.8%  *F*-measure.

### 8.2. PASCAL CHiME

In the first column of Table 2 we report the performance obtained on the PASCAL dataset using different approaches. Parts of the results obtained on this database were also presented in [36]. Here, we conducted additional experiments to evaluate OCSVM and MLP approaches. The one-class SVM shows lower performance compared to probabilistic approaches such as GMM and HMM, which seems to work reasonably well up to 91.4%  *F*-measure. The OCSVM low performance can be due to the fact that the dataset was generated artificially and the abnormal sound dynamics were normalized with respect to the “normal” material making the margin maximisation more complex and less effective. Next, we evaluated AE-based approaches in the three configurations: compression (CAE), basic (AE), and denoising (DAE). We also evaluated MLP, LSTM, and BLSTM unit types. Among the three configurations we observe that denoising ones perform better than the others independently of the type of unit. In particular, the best performance is obtained with the denoising autoencoder realised as BLSTM RNN showing up to 93.4%  *F*-measure. The last three groups of rows in Table 2 show results of the NP approach again in the three configurations and with the three unit types.

The NP-BLSTM-DAE achieved the best result of up to 94.4%  *F*-measure. Significant absolute improvements of 4.0%, 3.0%, and 1% are observed over GMM, HMM, and “ordinary” BLSTM-DAE approaches, respectively.

Interestingly, applying the nonlinear prediction scheme to the compression autoencoders NP-(B)LSTM-CAE (92.8%  *F*-measure) also increased the performances in comparison with the (B)LSTM-CAE (91.3%  *F*-measure). In fact, in a previous work [35], the compression learning process alone showed scarce results. However, here the CAE with the nonlinear prediction encodes information on the input more effectively.

**Figure 5:**
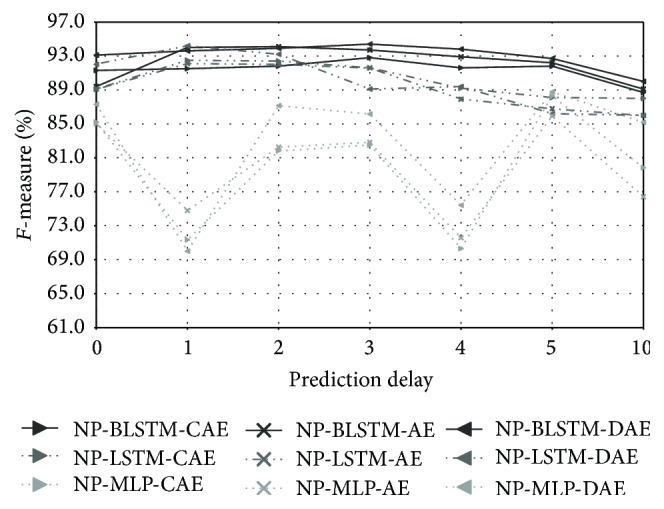
PASCAL CHiME: performances obtained with NP-Autoencoders by progressively increasing the prediction delay.

Figure 5 depicts results for increasing values of the prediction delay (*k*), ranging from 0 to 10. We evaluated CAE, AE, and DAE with MLP, LSTM, and BLSTM neural networks with different layouts (cf. Section 7) per network type. However, due to space restrictions, we only report the best performances. Here, the best performances are obtained with a prediction delay of 3 frames (30 ms) for the NP-BLSTM-DAE network (94.4%  *F*-measure) and of one frame in the case of NP-LSTM-DAE (94.2%  *F*-measure). As in the A3Novelty database, we observe a similar decrease in performance down to 86.2%  *F*-measure when the prediction delay increases up to 10, which corresponds to 100 ms. In fact, applying a higher prediction delay (e.g., 100 ms) induces higher values of the reconstruction error in the presence of fast periodic events, which subsequently leads to an increased false detection rate.

### 8.3. PROMETHEUS

This subsection elaborates on the results obtained on the four subsets present in the PROMETHEUS database.

#### 8.3.1. ATM

The ATM scenario evaluations are shown in the third column of Table 2. The GMM and HMM perform similarly at chance level. In fact, we observe an *F*-measure of 50.2% and 52.0% for GMMs and HMMs, respectively. The one-class SVM shows slightly better performance of up to 60.2%. On the other hand, AE-based approaches in the three configurations—compression (CAE), traditional (AE), and denoising (DAE)—show a significant improvement in performance up to 19.3% absolute *F*-measure against the OCSVM. Among the three configurations we observe that DAE performs better independently of the type of network. In particular, the best performance considering the ordinary (without nonlinear prediction) approach is obtained with the DAE with a LSTM network leading to an *F*-measure of 79.5%.

The last three groups of rows in Table 2 show results of the nonlinear predictive approach (NP). The nonlinear predictive denoising autoencoder performs best up to 81.6%  *F*-measure. Surprisingly, the best performance is obtained using MLP units suggesting that for long events—as those contained in the ATM scenario (with an average duration of 6.0 s, cf.  Table 1)—memory-enhanced units such as (B)LSTM are not as effective as for shorter events.

A significant absolute improvement of 21.4%  *F*-measure is observed against the OCSVM approach, while an absolute improvement of 31.4%  *F*-measure is exhibited with respect to the GMM-based method. Among the two autoencoder-based approaches we report an absolute improvement of 1.0% between the, namely, “ordinary” and “predictive” structures. It must be observed that the performance presented in [31] are higher than the one provided in this article since the tolerance window used in that study was set to 1 s whereas here we aimed at a higher temporal resolution with a tolerance window of 200 ms which is suitable also for abrupt events.

**Figure 6:**
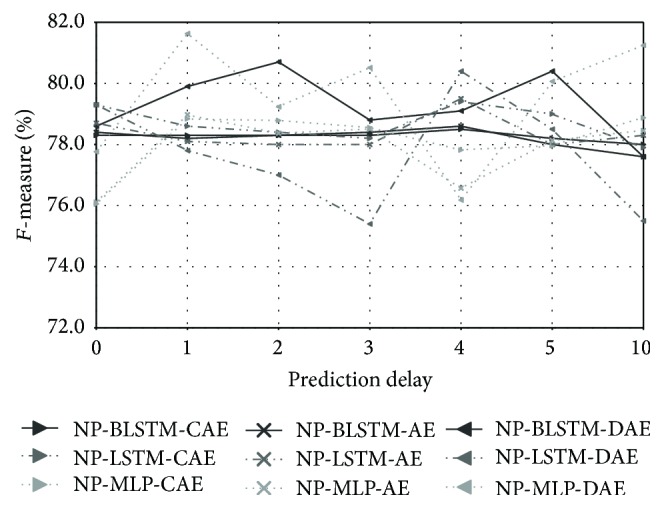
PROMETHEUS-ATM: performances obtained with NP-Autoencoders by progressively increasing the prediction delay.

Figure 6 depicts performance for progressive values of the prediction delay (*k*) ranging from 0 to 10, applying a CAE, AE, and DAE with MLP, LSTM, and BLSTM networks. Several layouts (cf. Section 7) were evaluated per network type; however, we report only the best configurations. Setting a prediction delay of 1 frame, which corresponds to a total prediction delay of 10 ms, leads to the best performance of up to 81.6%  *F*-measure in the NP-MLP-DAE network. In the case of the NP-BLSTM-DAE we observe better performance with a delay of 2 frames up to 80.7%  *F*-measure. In general, we do not observe a consistent trend by increasing the prediction delay, corroborating the fact that, for long events, as those contained in the ATM scenario, memory-enhanced units and a nonlinear predictive approach are not as effective as for shorter events.

#### 8.3.2. Corridor

The evaluations on the corridor subset are shown in the fourth column of Table 2. The GMM and HMM perform similarly at chance level. We observe an *F*-measure of 49.4% and 49.6% for GMM and HMM, respectively. The OCSVM shows better performance up to 65.3%. As observed in the ATM scenario, again a significant improvement in performance up to 16.5% absolute *F*-measure is observed using the autoencoder-based approaches in the three configurations (CAE, AE, and DAE) with respect to the OCSVM. Among the three configurations, we observe that the denoising autoencoder performs better than the others. The best performance is obtained with the denoising autoencoder with a BLSTM unit of up to 79.8%  *F*-measure.

The “predictive” approach is reported in the last three groups of rows in Table 2. Interestingly, the nonlinear predictive autoencoders do not improve the performance as we have seen in the other scenarios. A plausible explanation can be found based on the nature of the novelty events present in the subset. In fact, the subset contains very long events with an average duration of up to 14.0 s per event. With such long events, the generative model does not introduce a more sensitive reconstruction error. However, the delta in performance between the BLSTM-DAE and the NP-BLSTM-DAE is rather small (1.3%  *F*-measure) in favour of the “ordinary” approach. The best performance (79.8%) is obtained using BLSTM units confirming that memory-enhanced units are more effective in the presence of short events. In fact this scenario—besides very long events—also contains fall and pain short events with an average duration of 1.0 s and 3.0 s, respectively.

A significant absolute improvement up to 16.5%  *F*-measure is observed against the OCSVM approach, while being even higher with respect to the GMM and HMM.

#### 8.3.3. Outdoor

The evaluations on the outdoor subset are shown in the fifth column of Table 2. The OCSVM, GMM, and HMM perform better in this scenario as opposed to ATM and corridor. We observe an *F*-measure of 57.3%, 56.4%, and 56.0% for OCSVM, GMM, and HMM, respectively. In this scenario, the improvement brought by the autoencoder is not as vast as in the previous subsets but still significant. We report an absolute improvement of 11.2%  *F*-measure between OCSVM and BLSTM-DAE. Again, the denoising autoencoder performs better than the other configurations. In particular, the best performance obtained with BLSTM-DAE is 68.5%  *F*-measure.

As observed in the corridor scenario, the nonlinear predictive autoencoders (last three groups of rows in Table 2) do not improve the performance. These results corroborate our previous explanation that the long duration nature of the novelty events present in the subset affects the sensitivity of the reconstruction error in the generative model. However, the delta in performance between the BLSTM-DAE and NP-BLSTM-DAE is rather small (1.3%  *F*-measure).

It must be observed that the performance in this scenario is rather low compared to the one obtained in the other datasets. We believe that the presence of anger novel sounds introduces a higher degree of complexity in our autoencoder-based approach. In fact, anger novel events may contain different level of aroused content which could be acoustically similar to neutral spoken content present in the training material. Under this condition, the generative model shows a low reconstruction error. This issue could be solved by setting the novel events to only contain the aroused segments or—considering anger as a long-term speaker state—increasing the temporal resolution of our system.

#### 8.3.4. Smart-Room

The smart-room scenario evaluations are shown in the sixth column of Table 2. The OCSVM, GMM, and HMM perform better in this scenario as opposed to ATM, corridor, and outdoor. We observe an *F*-measure of 57.4%, 59.1%, and 59.1% for OCSVM, GMM, and HMM, respectively. In this scenario, the improvement brought about by the autoencoder is still significant. We report an absolute improvement of 6.0%  *F*-measure between GMM/HMM and the BLSTM-DAE. Again, the denoising autoencoder performs better than the other configurations. In particular, the best performance in the ordinary approach is obtained with the BLSTM-DAE of up to 65.1%  *F*-measure.

The last three groups of rows in Table 2 show results of the nonlinear predictive approach (NP). The NP-BLSTM-DAE performs best at up to 65.6%  *F*-measure.

As in the outdoor subset, we report a low performance in the smart-room subset as well. In fact, the subset contains several long novel events related to spoken content expressing pain and fear. As commented in the outdoor scenario, under this condition the generative model may be able to reconstruct the novel event without producing a high reconstruction error.

### 8.4. Overall

Overall, the experimental results proved that the DAE methods achieved superior performances compared to the CAE/AE schemes. This is due to the combination of two leaning processes of a denoising autoencoder, such as the process of encoding of the input by preserving the information about the input itself and simultaneously reversing the effect of a corruption process applied to the input of the autoencoder.

In particular, the predictive approach with (B)LSTM units showed the best performance of up to 89.3% average *F*-measure among all the six different datasets weighted by the number of instances per database (cf. Table 2).

**Figure 7:**
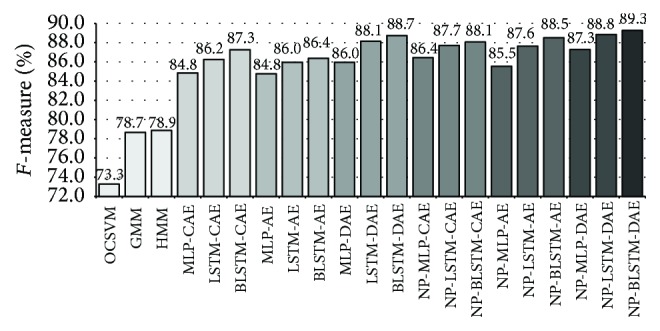
Average *F*-measure computed over A3Novelty Corpus, PASCAL, and PROMETHEUS weighted by the # of instance per database. Extended comparison of methods.

To better understand the improvement brought by the RNN-based approaches, we provide in Figure 7 the comparison between state-of-the-art methods in terms of weighted average *F*-measure computed across the A3Novelty Corpus, PASCAL CHiME, and PROMETHEUS. In general, we observe that the recently proposed NP-BLSTM-DAE method provided the best performance in terms of average *F*-measure of up to 89.3%. A significant absolute improvement of 16.0% average *F*-measure is observed against the OCSVM approach, while an absolute improvement of 10.6% and 10.4% average *F*-measure is exhibited with respect to the GMM- and HMM-based methods. An absolute improvement of 0.6% is observed over the “ordinary” BLSTM-DAE. It has to be noted that the average *F*-measure is computed including the PROMETHEUS database for which the performance has been shown to be lower because it contains long-term events and a lower resolution in the labels (1 s).

The RNN-based schemes also bring an evident benefit when applied to the “normal” autoencoders (i.e., with no denoising or compression); in fact, the NP-BLSTM-AE achieves an *F*-measure of 88.5%. Furthermore, when we applied the nonlinear prediction scheme to a denoising autoencoder, the performance achieved with LSTM was in this case comparable with BLSTM units and also outperformed state-of-the-art approaches.

In conclusion, the combination of the nonlinear prediction paradigm and the various (B)LSTM autoencoders proved to be effective, outperforming significantly other state-of-the-art methods. Additionally, there is evidence that memory-enhanced units such as LSTM and BLSTM outperformed MLP without memory, showing that the knowledge of the temporal context can improve the novelty detector abilities.

## 9. Conclusions and Outlook

We presented a broad and extensive evaluation of state-of-the-art methods with a particular focus on novel and recent unsupervised approaches based on RNN-based autoencoders. We significantly extended the studies conducted in [35, 36] by evaluating further approaches such as one-class support vector machines (OCSVMs) and multilayer perceptron (MLP), and most importantly we conducted a broad evaluation on three different datasets for a total number of 160153 experiments, making this article the first to present such a complete evaluation in the field of acoustic novelty detection. We show evidently that RNN-based autoencoders significantly outperform other methods by achieving up to 89.3% weighted average *F*-measure on the three databases, with a significant absolute improvement of 10.4% against the best performance obtained with statistical approaches (HMM). Overall, a significant increase in performance was achieved by combining the (B)LSTM autoencoder-based architecture with the nonlinear prediction scheme.

Future works will focus on using multiresolution features [51, 58], likely more suitable to deal with different event durations in order to face the issues encountered in the PROMETHEUS database. Further studies will be conducted on other architectures of RNN-based autoencoders ranging from dynamic Bayesian networks [59] to convolutional neural networks [60].

## Figures and Tables

**Table 1:** Acoustic novel events in the test set. Shown are the number of different events per database, the average duration, and the total duration in seconds per event type. The last column indicates the total number of events and total duration across the databases. The last line indicates the total duration in seconds of the test set including normal and novel events per database.

Events	A3Novelty		PASCAL CHiME		PROMETHEUS								Total	
					ATM		Corridor		Outdoor		Smart-room			
	#	Time (avg.)	#	Time (avg.)	#	Time (avg.)	#	Time (avg.)	#	Time (avg.)	#	Time (avg.)	#	time
Alarm	—	—	76	435.8 (6.0)	—	—	6	84.0 (14.0)	—	—	3	9.0 (3.0)	85	528.8
Anger	—	—	—	—	—	—	—	—	6	293.0 (48.8)	—	—	6	293.0
Fall	3	4.2 (2.1)	48	89.5 (1.8)	—	—	3	3.0 (1.0)	—	—	2	2.0 (1.0)	55	98.7
Fracture	1	2.2	32	70.4 (2.2)	—	—	—	—	—	—	—	—	33	72.6
Pain	—	—	—	—	—	—	2	8.0 (4.0)	—	—	5	67.0 (13.4)	7	75.0
Scream	6	10.4 (1.7)	111	214.6 (1.9)	5	30.0 (6.0)	25	228.0 (9.1)	4	48.0 (12.0)	10	234.0 (23.4)	159	762.2
Siren	3	20.4 (6.8)	—	—	—	—	—	—	—	—	—	—	3	18.1

Total	13	38.1 (2.9)	267	810.3 (3.1)	5	30.0 (5.0)	36	323.0 (9.0)	10	341.0 (34.1)	20	312.0 (15.6)	348	1848.4

Test time	—	5400.0	—	4188.0	—	750.0	—	960.0	—	1620.0	—	1020.0	—	13938.0

**Table 2:** Comparison over the three databases of methods by percentage of *F*-measure (*F*_1_). Indicated prediction (D)elay and average *F*_1_ weighted by the # of instances per database. Reported approaches are GMM, HMM, OCSVM, compression autoencoder with MLP (MLP-CAE), BLSTM (BLSTM-CAE), and LSTM (LSTM-CAE), denoising autoencoder with MLP (MLP-DAE), BLSTM (BLSTM-DAE), and LSTM (LSTM-DAE), and related versions of nonlinear predictive autoencoders NP-MLP-CAE/AE/DAE and NP-(B)LSTM-CAE/AE/DAE.

Method	A3Novelty		PASCAL CHiME		PROMETHEUS								Weighted average
					ATM		Corridor		Outdoor		Smart-room		
	D(*k*)	*F*_1_	D(*k*)	*F*_1_ (%)	D(*k*)	*F*_1_ (%)	D(*k*)	*F*_1_ (%)	D(*k*)	*F*_1_ (%)	D(*k*)	*F*_1_ (%)	*F*_1_ (%)
OCSVM	—	91.8	—	63.4	—	60.2	—	65.3	—	57.3	—	57.4	73.3

GMM	—	89.4	—	89.4	—	50.2	—	49.4	—	56.4	—	59.1	78.7

HMM	—	88.2	—	91.4	—	52.0	—	49.6	—	56.0	—	59.1	78.9

MLP-CAE	0	97.6	0	85.2	0	76.1	0	76.2	0	64.8	0	61.2	84.8
LSTM-CAE	0	97.7	0	89.1	0	78.5	0	77.8	0	62.6	0	64.0	86.2
BLSTM-CAE	0	98.7	0	91.3	0	78.4	0	78.4	0	62.1	0	63.7	87.3

MLP-AE	0	97.2	0	85.0	0	76.1	0	77.0	0	65.1	0	61.8	84.8
LSTM-AE	0	97.7	0	89.1	0	78.7	0	77.9	0	61.6	0	61.4	86.0
BLSTM-AE	0	97.8	0	89.4	0	78.5	0	77.6	0	63.1	0	63.4	86.4

MLP-DAE	0	97.3	0	87.3	0	77.5	0	78.5	0	65.8	0	64.6	86.0
LSTM-DAE	0	97.9	0	92.4	0	79.5	0	78.7	0	68.0	0	65.0	88.1
BLSTM-DAE	0	98.4	0	93.4	0	78.7	0	79.8	0	68.5	0	65.1	88.7

NP-MLP-CAE	4	98.5	5	88.3	1	78.8	2	75.0	1	65.2	5	64.0	86.4
NP-LSTM-CAE	5	98.8	1	92.5	1	78.7	2	74.4	2	64.7	3	63.8	87.7
NP-BLSTM-CAE	4	99.2	3	92.8	2	78.3	1	75.2	2	65.7	2	63.2	88.1

NP-MLP-AE	4	98.5	5	85.9	1	79.0	2	74.6	1	64.7	2	62.8	85.5
NP-LSTM-AE	5	98.7	1	92.1	1	78.1	1	75.0	1	65.0	3	64.4	87.6
NP-BLSTM-AE	4	99.2	2	94.1	3	78.4	2	75.6	1	65.6	1	63.6	88.5

NP-MLP-DAE	5	98.9	5	88.8	1	81.6	1	77.5	1	67.0	4	64.3	87.3
NP-LSTM-DAE	5	99.1	1	94.2	4	80.4	1	76.4	2	66.2	1	65.2	88.8
NP-BLSTM-DAE	5	99.4	3	94.4	2	80.7	3	78.5	2	66.7	1	65.6	89.3
